# Flavescence Dorée-Derived Leaf Yellowing in Grapevine (*Vitis vinifera* L.) Is Associated to a General Repression of Isoprenoid Biosynthetic Pathways

**DOI:** 10.3389/fpls.2020.00896

**Published:** 2020-06-17

**Authors:** António Teixeira, Viviana Martins, Sarah Frusciante, Telmo Cruz, Henrique Noronha, Gianfranco Diretto, Hernâni Gerós

**Affiliations:** ^1^Centre of Molecular and Environmental Biology, Department of Biology, University of Minho, Braga, Portugal; ^2^Centre for the Research and Technology of Agro-Environmental and Biological Sciences, University of Trás-os-Montes and Alto Douro, Vila Real, Portugal; ^3^Casaccia Research Center, ENEA, Italian National Agency for New Technologies, Energy and Sustainable Economic Development, Rome, Italy; ^4^Centre of Biological Engineering (CEB), Department of Biological Engineering, University of Minho, Braga, Portugal

**Keywords:** *Vitis vinifera*, flavescence dorée, biotic stress, leaf yellowing, isoprenoids biosynthesis, plant pathogens

## Abstract

Flavescence dorée (FD), caused by the phytoplasma *Candidatus* Phytoplasma vitis, is a major threat to vineyard survival in different European grape-growing areas. It has been recorded in French vineyards since the mid-1950s, and rapidly spread to other countries. In Portugal, the phytoplasma was first detected in the DOC region of ‘Vinhos Verdes’ in 2006, and reached the central region of the country in 2009. The infection causes strong accumulation of carbohydrates and phenolics in the mesophyll cells and a simultaneous decrease of chlorophylls, events accompanied by a down regulation of genes and proteins involved in the dark and light-dependent reactions and stabilization of the photosystem II (PSII). In the present study, to better elucidate the basis of the leaf chlorosis in infected grapevine cv. Loureiro, we studied the isoprenoid transcript–metabolite correlation in leaves from healthy and FD-infected vines. Specifically, targeted metabolome revealed that twenty-one compounds (out of thirty-two), including chlorophylls, carotenoids, quinones and tocopherols, were reduced in response to FD-infection. Thereafter, and consistently with the biochemical data, qPCR analysis highlighted a severe FD-mediated repression in key genes involved in isoprenoid biosynthetic pathways. A more diverse set of changes, on the contrary, was observed in the case of ABA metabolism. Principal component analysis (PCA) of all identified metabolites clearly separated healthy from FD-infected vines, therefore confirming that the infection strongly alters the biosynthesis of grapevine isoprenoids; additionally, forty-four genes and metabolites were identified as the components mostly explaining the variance between healthy and infected samples. Finally, transcript–metabolite network correlation analyses were exploited to display the main hubs of the infection process, which highlighted a strong role of *VvCHLG*, *VvVTE* and *VvZEP* genes and the chlorophylls intermediates aminolevulunic acid and porphobilinogen in response to FD infection. Overall, results indicated that the FD infection impairs the synthesis of isoprenoids, through the repression of key genes involved in the biosynthesis of chlorophylls, carotenoids, quinones and tocopherols.

## Introduction

Flavescence dorée (FD) has been recorded in French vineyards since the mid-1950s (Boudon-Padieu, 2002), and rapidly spread to other countries (Bertaccini et al., 2014). Nowadays, the FD has become a major threat to vineyard survival in different European grape-growing areas (Boudon-Padieu, 2002; Chuche and Thiéry, 2014; Margaria et al., 2014; Eveillard et al., 2016; Prezelj et al., 2016). In Portugal, FD was first detected in the Portuguese DOC region of ‘Vinhos Verdes’ in 2006 and the phytoplasma has reached the central region of the country since 2009 (https://www.drapc.gov.pt/).

FD is caused by the phytoplasma *Candidatus* Phytoplasma vitis, which is transmitted by the phytophagous insect *Scaphoideus titanus* Ball (Cicadellidae family). This vector is perfectly suited to southern European wine-growing areas, where summer is long enough for adults to lay their eggs (Bressan et al., 2005; Marzorati et al., 2006). Disease symptoms appear in early summer and increase in incidence and severity until harvest (Margaria et al., 2007; Abbà et al., 2014).

Different studies have reported the harmful effects of the infection at both physiological and molecular levels, revealing modifications in the primary and secondary metabolism. More in detail, it has been well established that the infection causes strong accumulation of carbohydrates and phenolics and decrease of chlorophylls. Accumulation of sucrose and starch in the leaf mesophyll is accompanied by the up regulation of key genes involved in their synthesis, including *Sucrose synthase 4* (*VvSusy4*) (Hren et al., 2009; Margaria et al., 2014; Prezelj et al., 2016). Likewise, a strong accumulation of proanthocyanidins and anthocyanins was detected in infected cv. ‘Barbera’ leaves, and the steady-state transcript levels of genes of the flavonoid pathway and proanthocyanidin branches were higher in infected plants (Hren et al., 2009; Margaria et al., 2014; Prezelj et al., 2016).

A hallmark of the infection by the grapevine diseases FD or Bois Noir (BN), is the reduction in chlorophyll content, resulting in leaf chlorosis, along with yellowing or reddening symptoms, depending on berry color, and lower net photosynthesis, together with a gradual decrease of transpiration (Bertamini and Nedunchezhian, 2001; Vitali et al., 2013; Oliveira et al., 2020). Photosynthesis is the main contributor of redox equivalents and energy fuelling biosynthesis of carbohydrates, amino acids and secondary metabolites in plants (Allahverdiyeva et al., 2015). Due to the redox chemistry, protection of the photosynthesis machinery from photodamage is one of the vital tasks performed by chloroplasts. Tocopherols, in cooperation with the xanthophyll cycle, act preserving PSII from photoinactivation and protecting membrane lipids from photooxidation (Kanehisa and Goto, 2000; Havaux and García-Plazaola, 2014; Allahverdiyeva et al., 2015). Also, isoprenoids like ubiquinone (UQ), and plastoquinone (PQ) are fundamental electron carriers acting in oxidative phosphorylation and photosynthesis, respectively (Ducluzeau et al., 2012). Besides their vital roles in photoprotection and as free radical scavengers, carotenoids are the precursors of several physiologically important apocarotenoids (Bouvier et al., 2005; Auldridge et al., 2006), like the phytohormone abscisic acid (ABA), originating from 9-cis-violaxanthin and -neoxanthin (Schwartz et al., 1997; Qin and Zeevaart, 2002).

In previous works, proteome analysis of cv. ‘Nebbiolo’ leaves revealed the down regulation of proteins involved in the photosystem II (PSII) stabilization e.g. Mn-stabilizing protein (Margaria and Palmano, 2011) and in infected leaves of cv. ‘Barbera’, proteins involved in the dark and light-dependent reactions of PS II were also less abundant (Margaria et al., 2013). Recent Next Generation Sequencing (NGS) analysis of *in vitro* micro propagated-infected Chardonnay plantlets showed that several genes involved in photosynthesis-related processes of both light and dark phases were down regulated (Bertazzon et al., 2019).

In the present study, to fulfill a gap in the literature, and as an attempt to better explain the observed leaf yellowing in response to FD-infection, we characterized metabolites and studied the expression of key genes involved in different isoprenoid pathways (chlorophylls, carotenoids, tocochromanols and quinones), by both untargeted and targeted metabolome and qPCR analyses. Leaves from FD-infected and control vines of cv. ‘Loureiro’ at two-snapshot time points were used. Loureiro is the second most cultivated variety in the Demarcated Region of Vinhos Verdes, the biggest Demarcated Region of Portugal, representing 15% of the viticultural area, with 192,000 ha and producing annually around 600 million L of wine (https://portal.vinhoverde.pt). Overall, besides chlorophylls, our work highlighted a large extent of alterations affecting the main isoprenoid classes.

## Material and Methods

### Plant Material

Grapevine leaves from the white vine ‘Loureiro’ cultivar, were collected in the 2018 season in a commercial vineyard of the Controlled Appellation (DOC) region of Vinhos Verdes in the northwest region of Portugal (41°31’01.0”N 8°12’56.1”W). This vineyard has a total area of 8 ha planted vines, and the sampling parcel has a northeast–southwest rows orientation. Vineyard rows were located on a steep hill with ca. 300 m altitude, and vines were managed without irrigation and grown using standard cultural practices as applied by commercial farmers.

Two sampling time points were chosen along the season: E-L 33 (leaves from vines at green stage) and E-L 38 (leaves from vines at mature stage). At each time point, four leaves from four infected vines (hereafter FD-infected) were collected from the sixth and eighth cane nodes. The diagnosis of infected vines was previously done by RFLP (Oliveira et al., 2020). Similarly, four leaves from four healthy control vines were collected. The leaves collected from each plant were pooled, transported to the laboratory in cooled containers and immediately frozen in liquid nitrogen before stored at −80 ˚C for posterior analysis. Leaves from each condition were grounded with liquid nitrogen to a fine powder, where half of powder was kept at −80°C for RNA extraction and the remain material was five days freeze dried in a Christ Alpha 2-4 LD Plus lyophilizer to be used in several biochemical quantification assays.

### Chlorophyll Quantification

Chlorophylls were extracted and quantified in healthy and infected leaves following the method of Lichtenthaler and Wellburn (1983) with minor modifications. Briefly, frozen dried leaf powder (50 mg) was mixed with 1.5 ml of methanol, toughly vortexed and kept for 24 h at 4°C in the dark. Samples were centrifuged at 14,000*×g*. The supernatant was recovered and the absorbance was measured at 666 nm, 653 nm and 470 nm wavelengths. Chlorophylls *a* and *b* were quantified using the following equations: C*a* = 15.65A_666_ – 7.34A_653_; C*b* = 27.05A_653_ – 11.21A_666_, where the different values correspond to methanol specific pigments absorption coefficients and *A* to the absorbance obtained in each wavelength.

### LC-DAD-HRMS Metabolomics

Identification and quantification of leaf isoprenoids were performed using liquid chromatography coupled to photodiode array and high-resolution mass spectrometry (LC-DAD-HRMS). More in details, extraction of atmospheric pressure chemical ionization (APCI) probe was used for the detection and the MS quantification of the nonpolar compounds (chlorophylls catabolites, tocochromanols and quinones), as reported in Sulli et al. (2017). Carotenoids were identified by APCI, and quantified based on the DAD peak intensities, as previously described (Rambla et al., 2016). Finally, polar compounds (chlorophyll precursors and intermediates) were extracted and analyzed using an electrospray ionization (ESI) source, as previously described (Fasano et al., 2016; D’Esposito et al., 2017). ABA and ABA catabolites detection and quantification were performed as reported in Diretto et al. (2020).

### RNA Isolation and qPCR Analysis

Total RNA was extracted from 200 mg of frozen ground leaf samples following the classical method described by Reid et al. (2006). For gene expression analysis by qPCR, total mRNA was converted to cDNA by reverse transcription with an Xpert cDNA Synthesis Kit and oligo (dT) primers (Grisp Research Solutions). Quantitative real-time PCR (qPCR) was performed in 96-well plates with Xpert Fast SYBR mastermix (Grisp Research Solutions). Briefly, for each biological condition (*n* = 3), qPCR reactions were performed in triplicate (technical replicates) using 10 μl MasterMix, 300 nM of each primer, 1 μl of cDNA and nuclease-free water to a final volume of 20 μl. The following cycler conditions were used: 15 min. at 95°C and 45 cycles of 15 s at 95°C, 30 s at 55°C and 30 s at 72°C. The sequences of gene-specific primers were designed from each gene sequence retrieved from KEGG: Kyoto Encyclopedia of Genes and Genomes database. When multiple gene isoforms for the same enzyme were present, the plastidial (methylerythritol 4-phosphate (MEP) pathway) one was selected (for more details, see statistical analysis and bioinformatics section). Gene expression was normalized to the *Glyceraldehyde 3-phosphate dehydrogenase* (*VvGAPDH*) reference gene (NCBI/Genbank database accession no. XM_002263109, (Gainza-Cortés et al., 2012). All primers sequences are detailed in **Supplementary Table S1**.

The specificity of PCR reactions was checked through dissociation curves at the end of each qPCR reaction, by heating the amplicons from 65 to 95°C. Data were analyzed using the CFX Manager Software (Bio-Rad laboratories, Inc.).

### Quantification of Total Reducing Sugars

Reducing sugars were quantified with the dinitrosalicylic acid method (DNS) according to Miller (1959). Briefly, the sugars were extracted in 50 mg of DW in 1 ml of warm ultrapure water. The homogenate was vigorously vortexed for 1 min, and centrifuged during 5 min at 18,000*×g*. The supernatant was recovered and pellet extracted again. Both supernatants were pooled and the quantification of reducing sugars was performed by mixing 250 µl of sample with 250 µl of DNS solution and boiled for 2 min. After that time, 2.5 ml of H_2_O were added and cooled to room temperature. Reducing sugar levels were then determined at 540 nm using a glucose standard curve.

### Identification and Quantification of Leaf Sugars by HPLC-RI

Sugar extraction was carried out as described previously by Eyéghé-Bickong et al. (2012). Briefly, 50 mg of lyophilized powder tissue were mixed with 1 ml of ultrapure H_2_O and thoroughly vortexed. A similar volume of chloroform was added to the mixture, and the biphasic solvent was vortexed for 5 min, and then incubated at 50°C for 30 min with continuous shaking (1,400 RPM). After incubation, the samples were centrifuged at 14,500*×g* for 10 min at 4°C, and the supernatants were collected. The extracted sugars were then filtered with PTFE 0.2 μm filters, and quantified by HPLC-RI using a Rezex RCM-Monosaccharide Ca^2+^ (8%) column (Phenomenex) at a flow rate of 0.2 ml min^−1^ at 40°C, using water as the mobile phase. Sugar concentration of each sample was determined by comparison of the peak area and retention time with standard samples curves.

### Statistical Analysis and Bioinformatics

Global non polar and polar compounds for untargeted metabolomics analyses were retrieved as previously described (Dono et al., 2020) using the SIEVE software (v2.2, ThermoFisher Scientific, Waltham, MA, USA). Principal Component Analysis (PCA) of leaf metabolomics data was performed in R software version 3.6.1 using the FactoMineR package 1.4.1 (Lê et al., 2008), and PCA output visualization was achieved with Factoextra package v1.0.5 (Kassambara and Mundt, 2017). Heatmaps of leaves metabolomics were performed with the ComplexHeatmap package (v1.18.1) on Bioconductor v3.9 (Gu et al., 2016). Both PCA’s and heatmap values were normalized by using the R centre and scale functions.

Bar graphs were plotted in Prism 7^®^ (GraphPad Software, Inc.). Significant differences between healthy and flavescence dorée-infected leaves were determined using Student’s *t* test and marked with asterisks to denote the significance levels: *P ≤0.05; **P ≤0.01; ***P ≤0.001; ****P ≤0.0001. All data are presented as mean values ± standard deviation (SD) of four biological replicates in each assay, except for gene expression analyses (*n* = 3). Center and scaled data was visualized on a metabolic map using the MapMan software (Thimm et al., 2004).

In targeted transcriptomics, only genes coding for proteins expressed or putatively expressed in the chloroplast of grapevine, or other plant in the absence of functional studies in grapevine, were considered. Gene sequences were retrieved from Kyoto Encyclopedia of Genes and Genomes (KEGG) and National Center for Biotechnology Information (NCBI) databases. Bioinformatics tool PSORT prediction (http://psort.hgc.jp/form.html) was used to predict the protein expression localization in the absence of functional studies in grapevine.

For network analysis, a correlation was assembled using MetScape plugin from Cytoscape version 3.7.2 (www.cytoscape.org; Cline et al., 2007), from a matrix of metabolites and transcripts values of FD-infected vines normalized on the corresponding controls, significantly different in at least one developmental stage, where the non-significant values (compared to control) in a particular condition were replaced by the value 1 (**Supplementary Table S2**), as reported in Diretto et al. (2010); Gómez-Gómez et al. (2017) and Aversano et al. (2017). In the network diagram, generated using a compound spring layout algorithm, edge thickness is proportional to the Pearson correlation values (|*ρ*|). Direct (*ρ* >0.70) and inverse (*ρ* ≤0.70) correlations were shown in red and blue, respectively. Different node shapes and colors were used to distinguish genes from metabolites. Finally, in order to better comprehend the FD infection process, we used the MCODE plugin, to obtain a series of metrical topological parameters of the network (density, cluster extrapolation etc.), which may allow, on mathematical basis, to infer biological information on transcript-metabolite cross-links (Bader and Hogue, 2003).

## Results

### Polar and Non-Polar Total Metabolome Changes in Response to FD Infection

**Figure 1:**
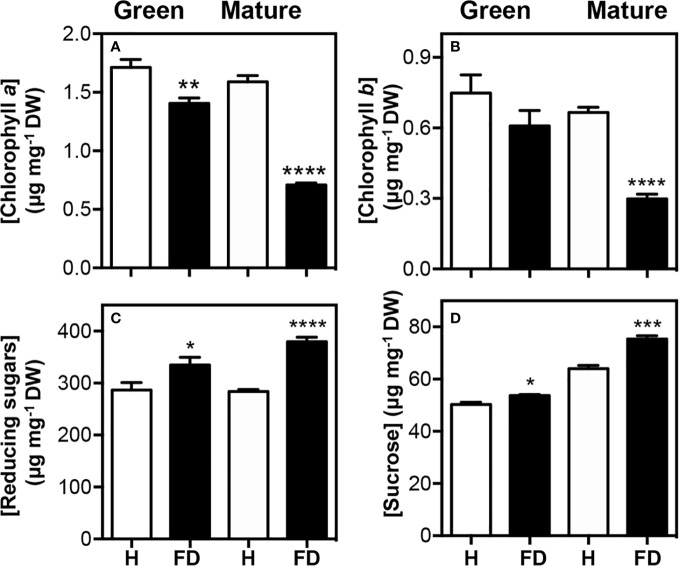
Primary metabolism compounds in leaves of healthy (H) and flavescence dorée-infected (FD) vines cv. ‘Loureiro’, collected in two grape berry developmental stages (green and mature). **(A)** chlorophyll *a*; **(B)** chlorophyll *b* levels; **(C)** reducing sugars and **(D)** sucrose levels. Results indicate mean ± SD of values of three biological replicates. Asterisks indicate statistical significance between FD-infected and the corresponding control (healthy) within each sample type following the Student’s *t*-test: *P ≤ 0.05; **P ≤ 0.01; ***P ≤ 0.001; ****P ≤ 0.0001.

In leaves from vines of cv. ‘Loureiro’ at the green stage, a statistically significant reduction was observed only for chlorophyll *a* in FD-infected plants compared to the control as measured by a spectrophotometric method, while at the mature stage, chlorophylls *a* and *b* contents were strongly reduced by ca. 55% in FD-infected plants compared to the control (**Figures 1A, B**), confirming previous results in the same cultivar obtained with a portable chlorophyll meter (SPAD meter) (Oliveira et al., 2020). The total reducing sugars in leaves were 14% (at the green stage) and 25% (at the mature stage) higher in FD-infected vines than in control plants (**Figure 1C**), but at the mature stage glucose only increased by 5% respectively, as measured by HPLC (not shown), while no differences were observed at the green stage between leaves from FD-infected and control vines. At green and mature stages, the levels of sucrose were 6 and 15% higher in leaves from FD-infected plants than in asymptomatic plants (**Figure 1D**).

**Figure 2:**
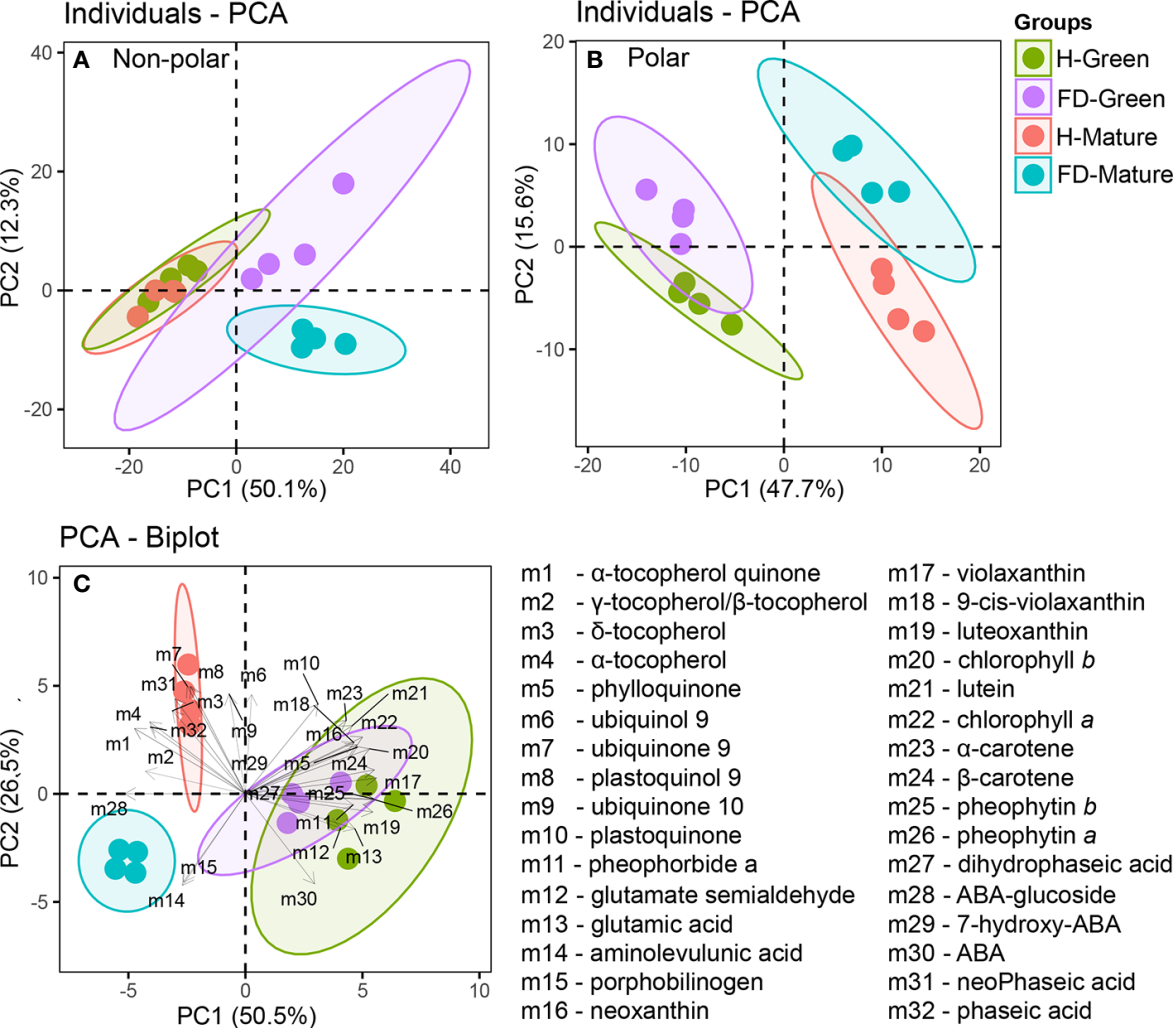
Principal Component Analysis (PCA) of untargeted and targeted metabolites measured in leaves of healthy (H-green and red dots) and flavescence dorée-infected (FD-violet and blue dots) vines cv. ‘Loureiro’, collected in two grape berry development stages (green and mature). **(A)** untargeted nonpolar metabolome; **(B)** untargeted polar metabolome and **(C)** BiPlot of chlorophyll precursors, chlorophylls, carotenoids, tocochromanols, quinones and ABA and ABA catabolites. Biological replicates in the score plots are shown with the same color within each sample condition and developmental stage, and the length of the arrows associated to each compound is proportional to its contribution to the overall sample distribution.

LC-DAD-ESI/APCI-HRMS analyses were performed to determine accurately the effect of flavescence dorée on grapevine leaf metabolites. An untargeted analysis was used to maximize the number of metabolites detected, and targeted analysis detected a relatively small number of metabolites, but already chemically characterized and biochemically annotated with established biological importance. Untargeted analysis revealed 366 compounds in the non-polar fraction and 221 in the polar fraction (**Supplementary Tables S2** and **S3**), whereas targeted analysis identified 32 metabolites. To construct the Principal Component Analysis (PCA) of **Figure 2**, all data from untargeted and targeted analysis were considered (**Supplementary Tables S2–S4**). PCA plot of non-polar fraction represented 62% of the total variation on the two first components. The PCA principal component 1 (PC1-*x* axis) contributed for 50% of the variability, separating clearly healthy from FD-infected samples (**Figure 2A**). The PCA principal component 2 (PC2-*x* axis) contributed for 12% of the variability, separating clearly FD-infected samples by developmental stage but not the healthy samples by developmental stage (**Figure 2A**).

PCA plot of polar fraction represented 63% of the total variation on the two first components and clearly separated the green from mature stages and healthy from infected vines (**Figure 2B**). The PCA principal component 1 (PC1-*x* axis) contributed for 48% of the variability and separate samples between developmental stages. The PCA principal component 2 (PC2-*y* axis) contributed for 16% of the variability and separate samples between phytosanitary conditions, with 60 measured metabolites correlated with PC2. Overall, FD-infection significantly reduced the non-polar fraction in leaves from vines at the mature stage, while affected in a lesser extent the polar fraction in leaves from vines at both developmental stages.

Targeted analyses were then performed to determine accurately the levels of fundamental non-polar compounds (isoprenoids and their intermediates) in leaf metabolism. The metabolites studied included chlorophylls, chlorophyll precursors, like glutamic acid and porphobilinogen, and chlorophyll catabolites, as pheophytin *a* and *b* and pheophorbide *a*; tocochromanols, like α- and β-/γ-tocopherol; quinones, as phylloquinone, plastoquinol-9 and ubiquinone-9 and 10; carotenoids, as ϵ-β and β-β-xanthophylls; ABA and its catabolites, as 7-hydroxy-ABA, phaseic acid and dihydrophaseic acid, for a total of thirty-two compounds (**Supplementary Table S4**). The PCA-Biplot of all identified metabolites showed a clear separation between leaves from FD-infected and healthy plants at mature stage contrary to green stage (**Figure 2C**). The first principal component (PC 1) accounted for 51% of variability, separating the samples according the development stage. Violaxanthin, β-carotene and pheophytin *b* contributed with the highest positive correlation values (above 0.95, *P <*0.000) and ABA-glucoside, γ-tocopherol/β-tocopherol and α-tocopherol-quinone with highest negative correlation (below −0.74, *P <*0.000). The second component (PC 2) accounted for 27% of variability, separating the samples from mature stage according to grapevines phytosanitary condition. Ubiquinone-9 and plastoquinol-9 contributed with the highest positive correlation values (above 0.86, *P <*0.000), and ABA and aminolevulunic acid contributed with the highest negative correlation (below −0.71, *P <*0.000) (**Figure 2C**).

**Figure 3:**
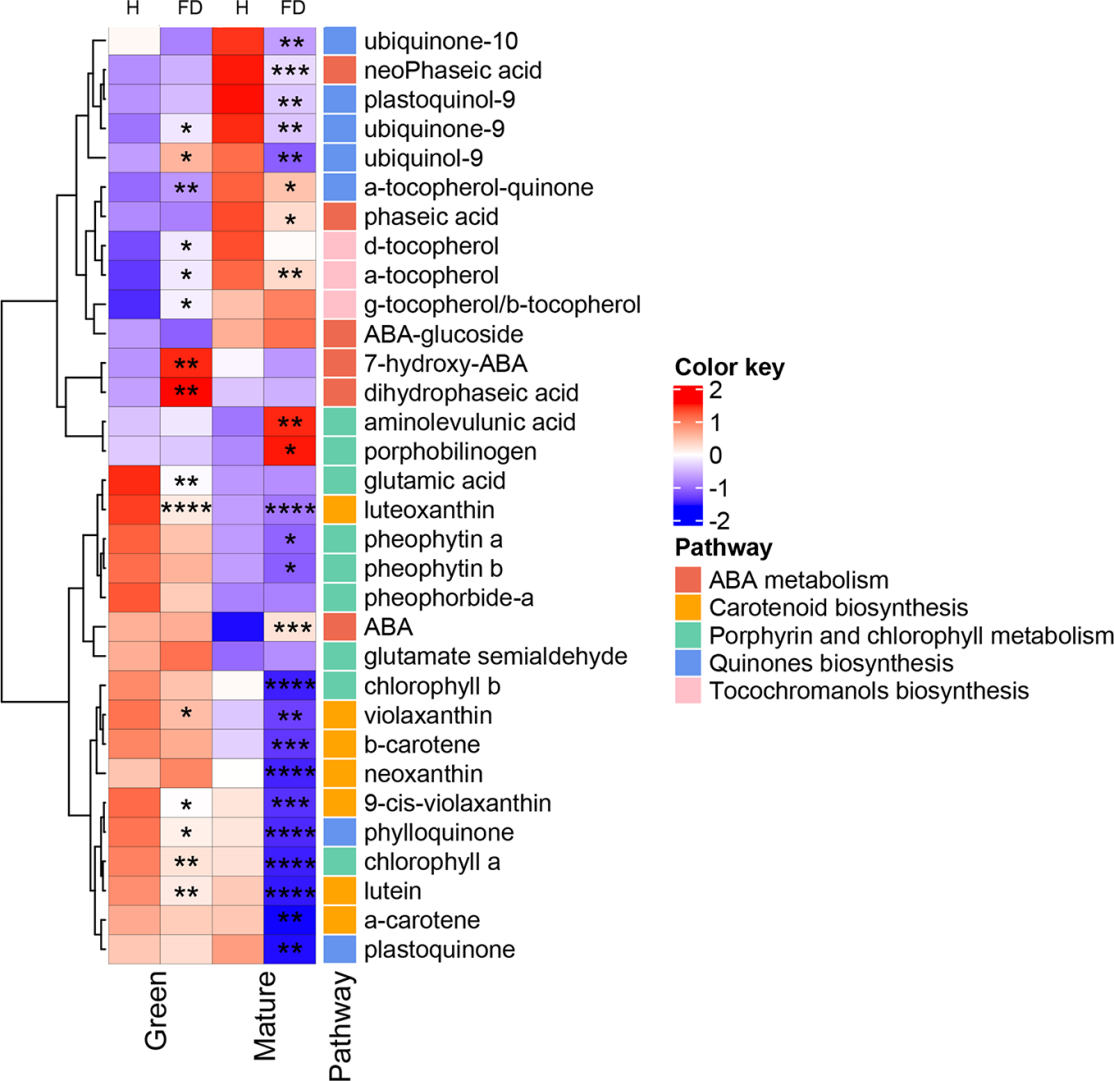
Heatmap of the modifications observed in chlorophyll precursors, chlorophylls, tocochromanols, quinones, carotenoids and ABA compounds in leaves of healthy (H) and flavescence dorée-infected (FD) vines cv. ‘Loureiro’, collected in two grape berry development stages (green and mature). Each row represents a metabolite and each column represents a sample development stage. Values were centered and scaled in the row direction to form virtual colors as presented in the color key, in which the offset was determined by the average values found within the four biological replicates of each sample type, and the scaling was defined according to the corresponding standard deviation. Metabolites were labeled according to their position in the biosynthetic pathways. Asterisks indicate statistical significance between FD-infected and the respective control (H-healthy) within each sample type following Student’s t-test: *P ≤0.05; **P ≤0.01; ***P ≤0.001; ****P ≤0.0001.

The heatmap of **Figure 3** was constructed with the data of the **Supplementary Table S4** and clusters hierarchically the compounds identified according mostly to their biosynthetic pathways in KEEG database with Vitis vinifera as reference. Isoprenoid biosynthetic pathways included five main categories: carotenoid biosynthesis, ABA metabolism, porphyrin and chlorophyll pathway, ubiquinone biosynthesis, and tocochromanols metabolism. Interestingly, hierarchical clustering of the rows, showed isoprenoids of the same metabolic class grouped together: indeed, from the top, it was clearly identified compounds taking place in quinone, tocochromanol, ABA, chlorophyll and carotenoid metabolism, with few exceptions.

Glutamic acid, the key early precursor of chlorophyll biosynthesis, showed a significant decrease (by 36%) in leaves from control to FD-infected vines at the green stage, but did not change in leaves from vines at the mature stage in response to infection (**Figure 3**). Glutamate semialdehyde, which appears two reactions ahead in the biosynthetic pathway of chlorophylls, displayed an apparent but not significant increase in FD-infected leaves at green and mature stages. In agreement, the intermediates aminolevulinic acid and porphobilinogen increased by 36% and 52%, respectively, in leaves from FD-infected vines at the mature stage but not at green stage.

In line with the results of **Figure 1**, HPLC-DAD analysis showed that both chlorophyll *a* and *b* decreased by 64 and 54%, respectively, in leaves from FD-infected vines at the mature stage, while at the green stage only chlorophyll *a* significantly decreased (by 22%). Both pheophytin *a* and *b*, derived by the partial degradation of chlorophylls, decreased by 16 and 29%, respectively, in leaves from FD-infected vines at the mature stage. Similar tendency was observed at green stage, although the decrease was not significant (**Figure 3**).

HPLC-DAD (**Figure 3** and **Supplementary Table S4**) data also revealed that most of the identified carotenoids decreased in leaves from FD-infected vines. At the green stage, luteoxanthin and lutein suffered a strong decrease, by 50 and 21% respectively. At the mature stage, leaves from FD-infected vines contained also less carotenoids than leaves from healthy plants, particularly luteoxanthin, α-carotene and lutein that decreased 68, 56 and 65%, respectively.

**Figure 3** also shows that at mature stage the ABA precursor β-β-xanthophylls, e.g. violaxanthin and neoxanthin, significantly decreased by 66 and 84% respectively, while at green stage only neoxanthin shows a significant decrease by 20%. At the mature stage, ABA levels were 77% higher in leaves from FD-infected vines than in controls, while at the green stage no differences in ABA levels were observed. At the green stage, the ABA catabolites 7-hydroxy-ABA and dihydrophaseic acid were 64 and 56% higher, respectively, in leaves from FD-infected vines than in controls, while at the mature stage the catabolites phaseic acid and neoPhaseic acid decreased by 24 and 44%, respectively, from control leaves to leaves from FD-infected vines (**Figure 3**, **Supplementary Table S4**).

Tocochromanols were also significantly affected by FD infection. At the green stage, indeed, the levels of three identified tocopherols increased in leaves from FD-infected plants, particularly α- and δ- forms (by 18 and 25%, respectively), although, very low amounts of δ- forms were detected in our experimental conditions. Contrary, at mature stage the δ-tocopherol form showed a significant decrease (by 23%). Quinones were also significantly affected by FD infection.

At the green stage, both ubiquinol-9 and ubiquinone-9 significantly increased by 25 and 16% respectively, in leaves from control to infected vines. Contrarily, at the mature stage, the seven identified quinones were significantly reduced upon FD infection: this group included α-tocopherol-quinone (19%), ubiquinone-9 (29% reduction), ubiquinone-10 (14% reduction), plastoquinone (22% reduction) and phylloquinone (50% reduction) (**Figure 3**, **Supplementary Table S4**).

### Expression of Target Genes Involved in Isoprenoid Biosynthesis in Response to FD-Infection

**Figure 4:**
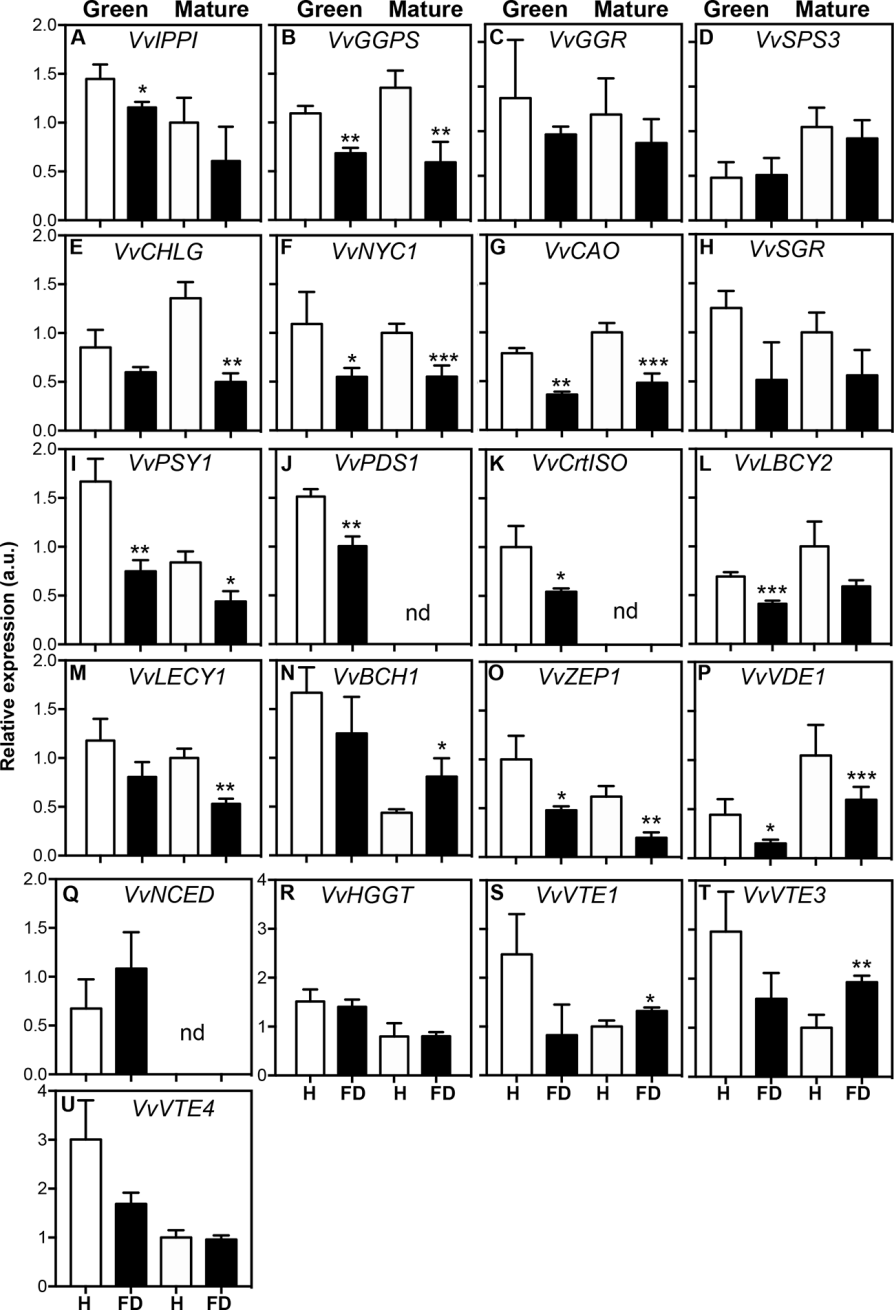
Expression of genes involved in isoprenoid biosynthesis in leaves of healthy (H) and flavescence dorée-infected (FD) vines cv. ‘Loureiro’, collected in two grape berry development stages (green and mature). **(A–D)** genes involved in isoprenoid precursor biosynthesis; **(E–H)** genes involved in chlorophyll biosynthesis and degradation; **(I–Q)** genes involved in the biosynthesis of carotenoids and ABA precursors; **(R–U)** genes involved in the biosynthesis of tocochromanols. Gene expression was normalized to the transcript levels of GAPDH (internal standard). Results indicate mean ± SD of values of three biological replicates. In bars, asterisks indicate statistically significant differences by the Student’s t-test: *P ≤ 0.05; **P ≤ 0.01; ***P ≤ 0.001, nd, non-detected.

**Figure 5:**
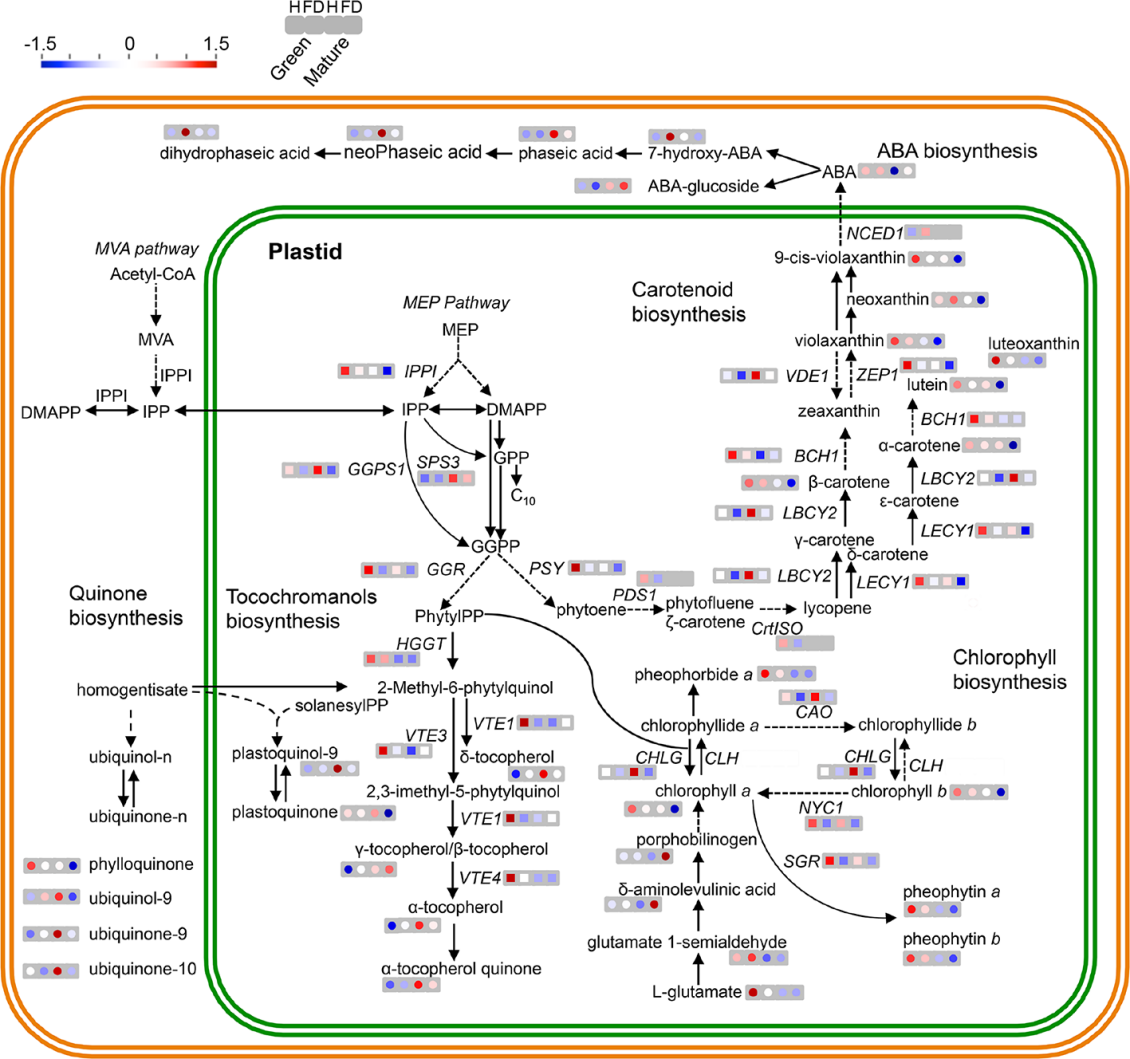
MapMan schematic representation of isoprenoid metabolic pathways in leaves of healthy (H) and flavescence dorée-infected (FD) vines cv. ‘Loureiro’. Simplified representation of metabolite biosynthesis steps and expression of key genes of the following pathways: MVA pathway in the cytosol or the methylerythritol 4-phosphate (MEP) pathway in plastids responsible for the synthesis of isoprenoid precursors isopentenyl diphosphate (IPP) and dimethylallyl diphosphate (DMAPP) and the expression of terpenoids backbone biosynthetic pathway genes *VvIPPI*, *VvSPS3*, *VvGGR* and *VvGGPS*; carotenoids biosynthetic pathway metabolites and the expression of *VvPSY*, *VvPSD1*, *VvCrtISO*, *VvLBCY2*, *VvLECY1*, *VvBCH1*, *VvVDE*, *VvZEP1* and *VvNCED1* genes; ubiquinone and other terpenoid-quinone biosynthetic pathway metabolites and the expression of *VvHGGT*, *VvVTE1*, *VvVTE3* and *VvVTE4* genes; porphyrin and chlorophyll biosynthetic pathway and the expression of *VvCHLG*, *VvCAO* and *VvNYC* genes. Metabolites content (circles) and transcript expression (squares) variation between healthy (H) and infected (FD) grapevines are represented by a colored scale: red—up regulated, blue—down regulated in both developmental stages (green and mature) from left to right, respectively. Grey squares represent a gene with no detected expression in that developmental stage or condition. Dashed arrows indicate multiple metabolic steps. Significant differences between FD-infected and respective controls are shown in **Figure 3** and **Supplementary Table S4**.

In order to determine if the observed biochemical changes reflected alterations at gene expression level, a set of target genes coding for enzymes of the isoprenoids pathway was studied by qPCR (**Figure 4**). The MapMan of **Figure 5** summarizes the results regarding gene expression and changes in metabolites of isoprenoid metabolic pathways in leaves of healthy and FD-infected vines. We firstly focused on transcripts involved in the production of the common isoprenoid blocks, most of them acting either in cytosolic mevalonate pathway (MVA pathway) from acetyl-CoA, and non-mevalonate pathway (MEP pathway) that occurs in the plastid (Nakamura et al., 2001). Results of **Figure 4** show that the steady-state transcript levels of *VvIPPI* were lower in leaves from FD-infected vines than in the control, albeit a statistically significant difference was only observed at the green stage, with a reduction of 25% (**Figure 4A**). Similarly, in leaves from vines at both the green and mature stages, the expression of *VvGGPS* suffered a significant reduction in leaves upon FD infection (**Figure 4B**). The average transcript levels of *VvGGR* were also slightly lower in leaves from FD-infected plants than in control although the values were not significantly different (**Figure 4C**). Finally, the expression of *VvSPS3* was not affected by FD infection (**Figure 4D**).

Subsequent studies were performed to evaluate the effect of FD infection in isoprenoids downstream pathways. The expression of key genes of the porphyrin and chlorophyll metabolism, carotenoids, tocochromanols and ubiquinone biosynthesis was then analyzed. Results showed that the steady-state transcript levels of *VvCHLG* were strongly lower (63% down regulation) in leaves from FD-infected vines than in control vines at the mature stage, while at the green stage the observed reduction was not statistically significant (**Figure 4E**). The expression of *VvNYC1* was down regulated by 50% in leaves from FD-infected vines at both the green and mature stages, and the expression of *VvCAO* followed the same expression pattern (**Figures 4F, G**). The average transcript levels of *VvSGR* were also lower in leaves from infected plants than in controls, but the differences were not significantly different (**Figure 4H**).

Transcript levels of *VvPSY1* were strongly reduced by up to 55% in leaves from FD-infected vines at both the green and mature stages (**Figure 4I**). The *VvPDS1* was down regulated by 34% in leaves from FD-infected vines at the green stage, while its expression was not detected at the mature stage in leaves from both FD-infected and control vines (**Figure 4J**). The *VvCrtISO* was down regulated by 46% in leaves from FD-infected vines at the green stage, while its expression was also not detected at the mature stage in leaves from both FD-infected and control vines (**Figure 4K**). The average steady-state transcript levels of both *VvLBCY2* and *VvLECY1* were also low in leaves from FD-infected plants compared to controls, albeit statistically significant differences were only observed at the green stage for the expression *VvLBCY2* (40% reduction), and at the mature stage for the expression of *VvLECY1* (47% reduction) (**Figures 4L, M**). In contrast, the *VvBCH* was up regulated upon FD infection, but only in leaves from vines at the mature stage (**Figure 4N**). The average transcript levels of *VvZEP1* with statistically significant differences were observed at the green and mature stages (52 and 68% down regulation respectively) (**Figure 4O**). The average transcript levels of *VvVDE1* were also lower in leaves from FD-infected vines than in control, but statistically significant differences were only observed at the green stage (65% down regulation) (**Figure 4P**).

The *VvNCED1* showed no significant differences in leaves from FD-infected vines at the green stage, while its expression was not detected at the mature stage in leaves from both FD-infected and control vines (**Figure 4Q**), which goes against the above-referred increase of ABA in at this stage in leaves from FD-infected vines.

The expression of other genes encoding for key proteins of the ubiquinone and other terpenoid-quinone biosynthesis pathway, including *VvHGG*, *VvVTE1*, *VvVTE3* and *VvVTE4*, was also studied, but their steady-state transcript levels not significantly changed upon FD-infection at green stage. Indeed, the average transcript levels of *VvVTE1* and *VvVTE3* increased in leaves from FD-infected vines at mature stage (**Figures 4R–U**, **Supplementary Table S4**).

### Transcript–Metabolite Correlation Is High in FD-Infected Leaves

**Figure 6:**
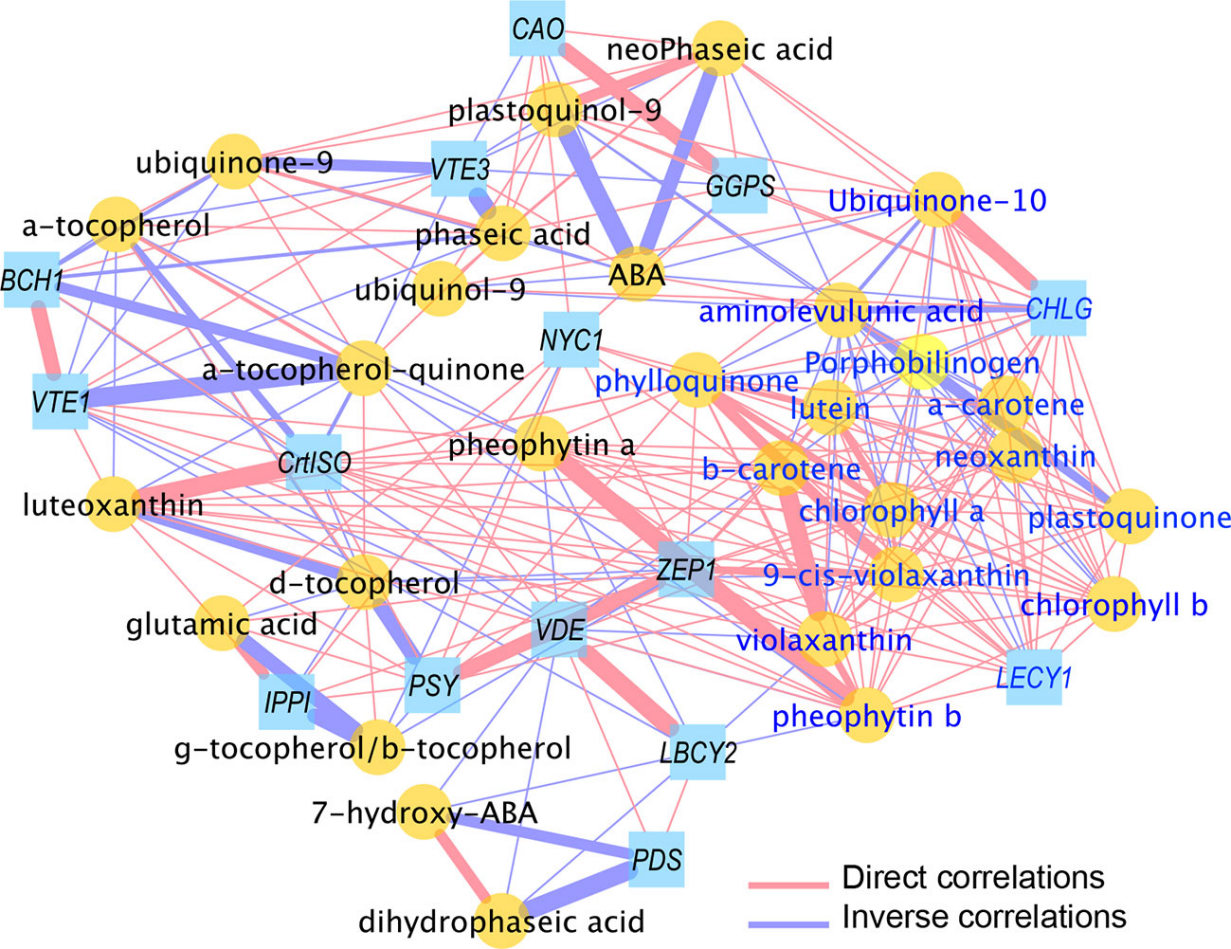
Correlation network of transcripts and metabolites from healthy (H) and flavescence dorée-infected (FD) grapevines cv. ‘Loureiro’ leaves collected at green and mature stage of grape berry development. Network is visualized with lines joining the nodes (edges) represent correlations (ρ>|0.70|); direct (positive) correlations are shown in red, while inverse (negative) correlations are shown in blue. Edge thickness is proportional to the respective correlation value (ρ). Node shapes represent a gene transcript (squares) or a metabolite (circles) from different biosynthetic pathways. For more details, see *Material and Methods* and **Supplementary Table S5**.

To further explore the relationships between transcript levels and metabolite contents, 44 variables significantly different in FD-infected grapevines leaves with respect to controls at both green and mature stages were chosen. The Pearson correlation coefficient values (|ρ|) for the resulting trait pairs were used to build a correlation network (Diretto et al., 2010). The overall ‘network strength’ was moderate (|ρ| = 0.52), indicating a moderate transcript-metabolite correlation in FD-infected leaves (**Figure 6**, **Supplementary Table S5**). A set of transcripts and metabolites traits grouped as a tight cluster in a region populated with compounds from porphyrin and chlorophyll metabolism, carotenoid biosynthetic pathway, and quinones biosynthesis (**Figure S1A**).

In detail, glutamic acid, the earliest precursor of chlorophylls, positively correlates with *VvIPPI*, *VvNYC1* and *VvPSY*, revealing a tight interaction between this metabolite and key genes of biosynthetic pathways. The chlorophyll intermediates aminolevulunic acid and porphobilinogen showed negative correlations with most of metabolites as carotenoids or quinones and gene transcripts as *VvGGPS*, *VvCHLG*, *VvLECY1* and *VvZEP1*. Interestingly, both intermediates positively correlate with ABA (ρ >0.77) but not with its catabolites. Besides phaseic and neoPhaseic acids, ABA is strongly negative correlated with quinone biosynthetic compounds (ρ ≤0.76) and with *VvGGPS*, *VvCHLG* and *VvCAO* genes but not with carotenoid pathway related genes (**Supplementary Table S5**).

Six of seven carotenoid metabolites present in this cluster positively correlated each other (ρ >0.91), with the gene *VvLECY1* playing a central position and strongly positively correlated with neoxanthin (ρ = 0.99), α-carotene (ρ = 0.99) or β-carotene (ρ = 0.97). Similarly, the transcript *VvZEP1* was strongly positively correlated with all seven carotenoids (ρ >0.86) and the transcript *VvPSY* with (9-cis-violaxanthin, luteoxanthin and lutein (ρ >0.80)) (**Supplementary Table S5**). Node strength (ns) calculation, intended as the average of all the |ρs| yielded by each node, was used to identify the main elements (“hubs” of the networks) involved in the FD infection (**Supplementary Table S5**): overall, a group of 10 hubs (mostly) metabolites were observed, which included chlorophyll a/b, several carotenoids (α-/β-carotene, lutein, violaxanthin, neoxanthin and *VvLECY1*) and phylloquinone.

Contrary of chlorophyll intermediates, chlorophylls and pheophytins positively correlates with all carotenoids and with quinones as phylloquinone or plastoquinone. Indeed, strong positive correlations were observed between chlorophylls and the *VvCHLG* gene and with carotenoid biosynthesis pathway genes (e.g. *VvLECY1* and *VvZEP1*) (**Figure 6**, **Supplementary Table S5**). Finally, negative correlations of ubiquinone and tocochromanols metabolites levels (α-tocopherol-quinone ubiquinol-9, ubiquinone-9, plastoquinol-9, α-tocopherol) were observed towards *VvVTE1* and *VvVTE3* genes involved in their synthesis, while metabolite-metabolite positive correlations of both phylloquinone and plastoquinone were also reported.

We used the MCODE Cytoscape plugin to better investigate the network topology, and to unravel the more dense areas, intended as the most crowded regions in terms of number of nodes (**Figure S1**). In addition, the MCODE algorithm was exploited to extrapolate, from the global network, transcript-metabolite clusters which are generated by weighting the |ns| and the ρ edges of each node towards the others; these are crucial parameters to identify groups of elements (nodes) showing the strongest relationships, thus potentially being the main interconnected players in the process under study. Interestingly, and in agreement with the previous data, a highly dense cluster 1 (**Figure S1A**) was found, which comprised nodes from each of the isoprenoid class under study (e.g. α-β-carotene, chlorophyll a/b, phylloquinone and ubiquinone-10); on the contrary, the remain subsequent identified clusters were characterized by a lower number of nodes (**Figures S1B–D**), thus indicating a lower contribution of the latters in the FD-induced grapevine infection.

## Discussion

### Leaves From FD-Infected cv. ‘Loureiro’ Show Higher Levels of Reducing Sugars

Different reports describe accumulation of proteins, sugars and other metabolites like phenolics in grapevine leaves in response to FD infection, caused by the blockage of the phloem vessels due to callose deposition in the sieve elements (Margaria et al., 2014; Prezelj et al., 2016; Oliveira et al., 2020). Likewise, in the present study, leaves from FD-infected cv. ‘Loureiro’ accumulated reducing sugars and sucrose. In coconut palms affected by lethal yellowing, phloem blockage is associated with an increase of starch in source leaves and a decrease in sink organs (Maust et al., 2003). Build-up of sucrose and starch in leaves of flavescence dorée infected vines is accompanied by the up regulation of key genes involved in their synthesis, including *VvSusy4* and *VvInv2* genes (Hren et al., 2009; Margaria et al., 2014; Prezelj et al., 2016). Bois noir (BN) cause similar effects on soluble sugars and total saccharides in cv. ‘Chardonnay’ (Bertamini and Nedunchezhian, 2001).

The strong modification in primary metabolism is associated with shifts in secondary metabolism when sugars feed the shikimate pathway and secondary metabolism. In addition, as discussed below, the accumulation of sugars in leaves may trigger modifications in photosynthetic metabolism at the transcriptional level.

### Leaf Yellowing in FD-Infected Vines Is Caused by General Reduction of Chlorophyll Biosynthesis Through a Transcriptional Reprogramming of Key Genes

A hallmark of FD is the development of leaf yellowing or reddening depending on berry color (de Sousa et al., 2010; Oliveira et al., 2019). This symptom was observed in leaves of FD-infected cv. ‘Loureiro’ associated to a strong decrease of both chlorophyll *a* and *b*, as measured by a conventional spectrophotometric method and HPLC. This reduction in chlorophyll content was recently reported in the same vines with a non-destructive method (SPAD meter) (Oliveira et al., 2020). Leaf yellowing was also previously observed in BN-infected Chardonnay (Bertamini and Nedunchezhian, 2001).

As a whole, we confirmed the hypothesis that leaf yellowing is associated caused by FD-mediated repression of the biosynthetic pathways of chlorophylls; however, an important novelty of the present study is that additional isoprenoid classes, carotenoids, tocochromanols and quinones, were also strongly negatively affected. In agreement with this result, we found that in leaves from FD-infected plants key genes involved in the biosynthesis of chlorophylls, carotenoids, tocochromanols and quinones were strongly repressed. Similarly, twenty-five isoprenoids (including final products and intermediates), out of thirty-two identified in leaves, were reduced in response to FD infection. However, the extent of metabolic changes overcame the sole isoprenoid metabolism, as revealed by the untargeted analyses of both non-polar and polar metabolomes. Principal component analysis (PCA) highlighted a different contribution by the metabolomics fractions under study: indeed, while global polar and targeted non-polar compounds separated the samples under study according the stage, global non-polar molecules allowed a grouping according to treatment. This is not surprising, since metabolic alterations can be affected at variable extent by the process of infection, the genetic background and its interaction with the environment (reviewed by Kirkwood et al., 2013; Ma et al., 2019; Dono et al., 2020). Additionally, the divergent behavior of the untargeted and targeted non-polar analyses suggest the existence, in the former, of additional compounds which are specifically affected by the FD-infection.

However, it is noteworthy mentioning that also targeted isoprenoid metabolomics clearly distinguish healthy from FD-infected vines, therefore confirming that the infection strongly impairs the biosynthesis of grapevine isoprenoids, with a primarily role of violaxanthin, β-carotene, pheophytin *b* and chlorophyll *b* as main contributors.

Thus, the steady state transcript levels of *VvIPPI*, involved in the synthesis of IPP isomerase, which catalyzes the conversion of Isopentenyl diphosphate (IPP) to dimethylallyl diphosphate to form the basic five-carbon isoprene unit for IPP condensation, was down regulated in leaves from FD-infected vines. Because IPP is the common precursor of all isoprenoids in cytosolic MVA biosynthesis and in non-mevalonate MEP pathway occurring in the plastids, its down regulation in leaves likely contributed to the general reduction of the intermediates and the end products of isoprenoid metabolic pathways. The same trend was followed by other key genes like *VvGGPS*, which encodes a geranylgeranyl diphosphate synthase that converts dimethylallyl diphosphate in geranylgeranyl diphosphate (GGPP). GGPP is a major branching point for several downstream terpenoids pathways. These include the biosynthesis of chlorophylls, carotenoids and their breakdown products (abscisic acid (ABA)), strigolactones (SLs)), tocochromanols, gibberellins, plastoquinones and diterpenoids, all synthesized in plastids (Tholl, 2015).

Contrarily to the central genes *VvIPPI* and *VvGGPS* of the isoprenoids biosynthesis, the average transcript levels of *VvGGR* and *VvSPS3* were not affected by FD-infection. The first encodes a geranylgeranyl reductase that converts geranylgeranyl diphosphate in phytyl diphosphate, which takes part in tocopherol and plastid quinone synthesis, while the second encodes a solanesyl diphosphate synthase converting the isopentenyl diphosphate to geranyl pyrophosphate (Kanehisa and Goto, 2000). Thus, our data suggest FD can trigger a direct and negative perturbation of transcripts acting as key master genes of the isoprenoid metabolism, which resemble previous reports on the general regulation of isoprenoid pathway (as reviewed in Vranová et al. (2012).

Results suggested that the synthesis of chlorophylls is regulated at transcriptional level because the steady-state transcript levels of *VvCHLG*, *VvCAO* and *VvNYC1* were reduced in FD-infected plants. Both chlorophyll synthase [CHLG—EC:2.5.1.62], and chlorophyllide *a* oxygenase [CAO—EC:1.14.13.122] catalyze the last steps of chlorophyll biosynthesis: chlorophyll *a* from chlorophyllide + phytyl diphosphate and chlorophyllide *a* to the *b* form, a step before chlorophyll *b* synthesis by CHLG (Kanehisa and Goto, 2000). Chlorophyll(ide) *b* reductase, [NYC1—EC:1.1.1.294] is involved in the first step conversion of chlorophyll *b* to chlorophyll *a* and in chlorophyll degradation (Hörtensteiner, 2006; Shimoda et al., 2016), while *VvSGR* codes for a STAY-GREEN 1 protein [SGR—EC:4.99.1.10] is required for the initiation of chlorophyll *a* breakdown into Pheophytin *a,b* (Sakuraba et al., 2012; Shimoda et al., 2016). Non-yellow coloring1 (nyc1) is a rice (*Oryza sativa*) stay-green mutant in which chlorophyll degradation during senescence is impaired (Kusaba et al., 2007). In the present study, *VvNYC1* was strongly repressed in leaves from FD-infected vines at the green and mature stage, while the expression levels of *VvSGR* was slightly repressed but not significantly. In parallel, results showed that the chlorophyll degradation products pheophytin *a* and *b*, were reduced in leaves from FD-infected vines. Thus, these results suggest that the observed leaf chlorosis in response to FD infection is due to a general decrease in the metabolic flux along the whole chlorophyll metabolism and catabolism, rather than to an increase in its degradation.

Some reports have suggested that the higher levels of free hexoses in plants could account for a feedback inhibition of photosynthesis (Sheen, 1990; Sheen, 1994), eventually through the repression of the expression of key genes, which could be the case of the present study. Other reports have already described the repression of specific metabolic pathways of the photosynthetic apparatus mediated by FD or BN infection. In BN-infected cv. ‘Chardonnay’, the down regulation of eleven genes encoding chlorophyll *a*/*b*-binding proteins in the PSII and three in PSI was observed, together with a group of transcripts involved in light reactions of photosynthesis that resulted in a serious inhibition of whole photosynthetic chain and photosystem I activity (Albertazzi et al., 2009; Hren et al., 2009).

At a first glance, the results also suggested that the observed strong reduction of L-glutamic, the early precursor of chlorophyll, could contribute, at least in part, for the observed reduction of chlorophyll *a*, but paradoxically, the polar intermediates phorphobilinogen and δ-aminolevulinic acid (ALA), universal precursors of tetrapyrroles, were more abundant in leaves from FD-infected vines. However, accordingly, it was suggested that a reduced activity of Chl Synthase could account for the accumulation of intermediates of *Chl* biosynthesis, including ALA (Wu et al., 2007). Furthermore, it has been reported that chlorophyll intermediates play a role as signalling molecules in response to abiotic and stress responses (Quesada et al., 2013; Anwar et al., 2018; reviewed by Wu et al., 2019). In this context, the higher accumulation of phorphobilinogen and δ-aminolevulinic acid (ALA) might be interpreted as an attempt of the grape leaves to counteract the infection process.

### Key Carotenoids Are Reduced in FD-Infected cv. Loureiro but ABA Levels Increase

In general, key genes of the carotenoid biosynthetic pathway, including *VvPSY1*, *VvPDS1*, *VvCrtISO*, *VvLBCY2*, *VvLECY1*, *VvBCH*, *VvVDE1*, *VvZEP1* and *VvNCED1*, suffered a reduction in leaves from FD-infected vines. *VvPSY1* and *VvPDS1* encode, respectively, a phytoene synthase converting two molecules of geranylgeranyl diphosphate to phytoene, and a phytoene desaturase utilizing phytoene to yield ζ-carotene. *VvCrtISO* codes for a carotene isomerase that converts 7,9,7′,9′-tetra-cis-lycopene in all-trans-lycopene, which acts as substrate of *VvLBCY2* and *VvLECY1*, two competing lycopene β-/ϵ-cyclase enzymes producing, respectively, *γ-/*β-, and *δ-/*ϵ-/α-carotenes. *VvBCH* codes a β-carotene 3-hydroxylase that converts β-carotene to zeaxanthin and *α*-carotene to zeinoxanthin, the lutein precursor; while *VvZEP1* and *VvVDE1*, encoding a zeaxanthin epoxidase and violaxanthin de-epoxidase, respectively, are the two components of the xanthophyll cycle, involved in the zeaxanthin to violaxanthin intercoversion to protect plant cells by light excess conditions (Kanehisa and Goto, 2000; Latowski et al., 2011).

Both *in vitro* and *in vivo* evidences demonstrate that phytochrome-interacting factor 1 (PIF1) directly binds to the promoter of the *PSY* gene, resulting in repression of the *PSY* expression (Toledo-Ortiz et al., 2010). More recently, it was proposed that the biosynthesis of carotenoids in Arabidopsis is controlled by the physical interaction of OR proteins (AtOR and AtOR-like) with PSY, being the major posttranscriptional regulators of *PSY* in plants (Zhou et al., 2015). The way the expression of these genes is regulated in response to FD seems a good topic for future research.

The rate-limiting step for ABA biosynthesis in leaves is believed to be the cleavage of 9-cis-epoxyxanthophylls by the NCED dioxygenase (Thompson et al., 2000; Qin and Zeevaart, 2002; Giuliano et al., 2003; Diretto el al., 2020). However, we observed that the expression of *VvNCED1* transcripts were not detected in leaves from both healthy and FD-infected grapevines at mature stage albeit in leaves from FD-infected plants the levels of ABA were high. This intriguing observation warrants further investigation, in particular to determine if the increased levels of ABA in FD-infected plants are produced in other plant tissues like roots (Hu et al., 2016) and to study the expression of other *VvNCED* genes that could, hypothetically, play a more important role in ABA production than *VvNCED1*. Indeed, in *Arabidopsis*, among the five *NCED* genes involved in ABA biosynthesis, only *AtNCED3* was highly induced by dehydration (Iuchi et al., 2001; Tan et al., 2003; Reviewed by Nambara and Marion-Poll, 2005). This thesis could be supported by the evidence that an up regulation of one *NCED* member has been detected by a transcriptomic approach on two Italian grapevine varieties (Chardonnay and T. friulano), although in this case no metabolite analyses on ABA have been carried out (Bertazzon et al., 2019).

Another hypothesis is that the ABA accumulation, as consequence of the FD infection that could mimic the senescence status, could be achieved by the existence of control mechanisms acting on transcript levels of downstream genes with respect to *NCED* (as already described by Bertazzon et al., 2019), or at post-transcriptional rather than at RNA level; this latter hypothesis would be supported by previous studies which have shown the existence of post-transcriptional regulation especially at ABA signaling gene level (Yang et al., 2017).

GGPP could also be channeled to the ubiquinone and other quinone biosynthesis by the action of different key enzymes depicted in **Figure 5**, but results showed that the expression of *VvVTE1*, *VvVTE3* and *VvVTE4* was not repressed in response to FD-infection, although different end products were reduced; they included plastoquinone and ubiquinone-9/-10, which act as carriers of electrons in the chloroplast thylakoids and mitochondrial inner membranes, respectively (Yoshida et al., 2010). Interestingly, plastoquinone also takes part in chlororespiratory and serves as component of phytoene desaturation in carotenoid pathway (Norris et al., 1995). Therefore, also the findings on tocochromanol and quinone accumulation might be explained as downstream effects of the general reduction of the early isoprenoid precursors.

### Transcript–Metabolite Isoprenoid Are Highly Correlated Upon FD-Infection

We exploited correlation network approach to better investigate the isoprenoid gene-metabolite relationships in leaves infected by FD, compared to healthy ones. The network strength (|ρ| = 0.52) suggests a well coordinate re-programming of the different isoprenoid classes at transcript–transcript, metabolite–metabolite and transcript–metabolite level upon FD-infection. This extent of coordination and conservation of related secondary metabolites has been already described in previous studies (Rambla et al., 2016; D’Esposito et al., 2017; Dono et al., 2020), and can be explained with a rapid and highly organized capacity of the plant cells to react against a biotic stressor.

However, the analysis of the network topology evidenced an upper located region with a larger number of significant correlations, highly abundant in carotenoid metabolites and late transcripts (*VvLECY1*, *VvZEP1* and, although more distal, *VvPSY*). Node spatial distribution is not random and was, indeed, generated using a compound spring layout algorithm, which locates the nodes according geometry derived by the overall relationships (ρ edges) that each node owns with all the others (Pavlopoulos et al., 2017; Diretto et al., 2020). For this reason, the presence of a carotenoid-enriched area indicates a central role for this compound class in the biochemical responses to FD. Furthermore, porphyrin metabolites and chlorophylls were also present in this region, thus confirming the decrease in chlorophyll expression and metabolite content are main events following the FD infection of grapevine leaves, while additional changes might occur as downstream effects according a sequential cascade. The MCODE Cytoscape plugin was exploited to achieve additional information on the transcript–metabolite relationships evolving upon FD-infection in the isoprenoid group, taking advantage of the possibility to identify group of nodes placed in highly interconnected (dense) regions according their correlation coefficient parameters (ρ edges) and defined clusters; originally, it was used for protein data (Cline et al., 2007), with the assumption that a protein dense region may suggest the existence of protein complexes. However, it has been subsequently exploited also for transcript and metabolite data (Zhang et al., 2019; Dono et al., 2020), in order to highlight potential core regulatory networks and candidate genes/metabolites in a specific process. In our data, and as evidenced by the MCODE Cytoscape plugin, a large cluster was found, mostly comprising elements involved in chlorophyll and carotenoid pathways, thus emphasizing their relevant role in the grapevine infection by FD. In agreement with this hypothesis, several metabolites of these classes displayed the highest node strength (ns) (measured as indicated in materials and methods and reported in Diretto et al., 2010; Diretto et al., 2020), indicating a coordinated and active role of these pathways in the responses to the FD-infection.

The extensive analysis of each pair correlation highlighted the presence of numerous negative correlations of ubiquinone and tocochromanols metabolites levels (α-tocopherol-quinone ubiquinol-9, ubiquinone-9, plastoquinol-9, α-tocopherol) against *VvVTE1* and *VvVTE3* genes, which are involved in their synthesis; this finding might suggest an attempt to counteract the decrease in these metabolites. Indeed, the higher expression levels of these genes at mature stage may indicate a physiologic response of infected plants, given the importance of this group of isoprenoids for PSI and PSII complex stability (Havaux and García-Plazaola, 2014; Wang et al., 2017). Finally, the observed metabolite–metabolite positive correlations of both phylloquinone and plastoquinone might provide clues about the existence of a metabolic coregulation that might be exacerbated by the infection.

## Conclusions

Here, we detail the effect of flavescence dorée on grapevine isoprenoid metabolism with a combined approach based on targeted metabolomics and qPCR analysis of key genes. This approach allowed shedding light on how FD infection affects core metabolic pathways, including MVA/MEP pathways, and chlorophylls, carotenoids, tocochromanols, quinones and ABA biosynthesis. In general, FD promoted the accumulation of carbohydrates and of two chlorophyll precursors in leaves, and repressed the synthesis of all the isoprenoid species analyzed, by down regulating key synthetic genes. Also, the strong down regulation of *VvIPPI*, *VvGGPS*, *VvPSY* and *VvLECY1* suggests that these are key genes involved in the observed reduction of carotenoid levels, like β-carotene lutein, and 9-cis-epoxyxanthophylls (violaxanthin and neoxanthin. Additionally, since the infection increased the number moderate and significant correlations between isoprenoid elements acting in a simultaneous or sequential way (transcript–transcript, metabolite–metabolite, transcript–metabolite), our data provide hints about the existence of a tight regulation at both transcriptional and posttranscriptional levels in the grapevine responses to the FD-infection.

## Data Availability Statement

All datasets presented in this study are included in the article/**Supplementary Material**.

## Author Contributions

AT, VM, HN, and HG conceptualized the work. AT and TC performed the field and laboratory sample processing. AT, VM, and TC performed the biochemical analysis and targeted transcriptomics. SF and GD performed metabolomic analysis and data treatment. AT, HG, and GD analyzed the results. AT, GD and HG wrote the manuscript. AT, VM, HN, GD, and HG reviewed the manuscript. All authors contributed to the article and approved the submitted version.

## Conflict of Interest

The authors declare that the research was conducted in the absence of any commercial or financial relationships that could be construed as a potential conflict of interest.
