# Supplementary material for: Flavescence Dorée-Derived Leaf Yellowing in Grapevine (Vitis vinifera L.) Is Associated to a General Repression of Isoprenoid Biosynthetic Pathways
Source: Front Plant Sci. 2020 Jun 17;11:896. doi: 10.3389/fpls.2020.00896 (PMC7311760; doi:10.3389/fpls.2020.00896)
Supplement: Supplementary file 2 [file Image_1.pdf]

Supplemental files

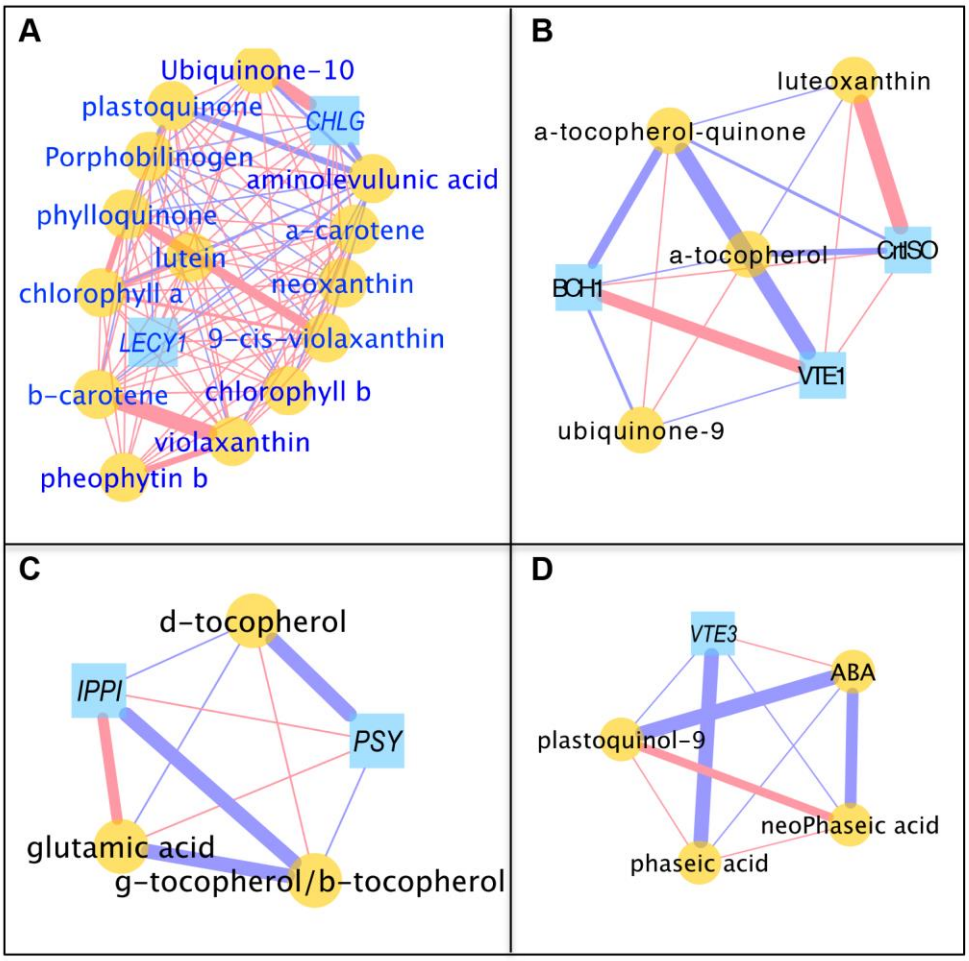

Supplemental Figure S1. Sub networks generated using the MCODE Cytoscape plugin applied to the global isoprenoid network of Figure 8, and ordered according the descending density (a: cluster 1; b: cluster 2; c-c: clusters 3). Each node represents a transcript or metabolite of one of the isoprenoid classes under study (chlorophylls, carotenoids, tocochromanols, quinones and ABA). Lines joining the nodes represent positive (red) and negative (blue) correlations. Only correlations with |ρ|>0.70 are shown (p-value 0.05).
